# Bioinformatic evidence for a stem-loop structure 5'-adjacent to the IGR-IRES and for an overlapping gene in the bee paralysis dicistroviruses

**DOI:** 10.1186/1743-422X-6-193

**Published:** 2009-11-06

**Authors:** Andrew E Firth, Qing S Wang, Eric Jan, John F Atkins

**Affiliations:** 1 BioSciences Institute, University College Cork, Cork, Ireland; 2 Department of Biochemistry and Molecular Biology, University of British Columbia, 2350 Health Sciences Mall, Vancouver, BC, Canada; 3 Department of Human Genetics, University of Utah, Salt Lake City, UT 84112-5330, USA

## Abstract

The family *Dicistroviridae *(order *Picornavirales*) includes species that infect insects and other arthropods. These viruses have a linear positive-sense ssRNA genome of ~8-10 kb, which contains two long ORFs. The 5' ORF encodes the nonstructural polyprotein while the 3' ORF encodes the structural polyprotein. The dicistroviruses are noteworthy for the intergenic Internal Ribosome Entry Site (IGR-IRES) that mediates efficient translation initation on the 3' ORF without the requirement for initiator Met-tRNA. Acute bee paralysis virus, Israel acute paralysis virus of bees and Kashmir bee virus form a distinct subgroup within the *Dicistroviridae *family. In this brief report, we describe the bioinformatic discovery of a new, apparently coding, ORF in these viruses. The ORF overlaps the 5' end of the structural polyprotein coding sequence in the +1 reading frame. We also identify a potential 14-18 bp RNA stem-loop structure 5'-adjacent to the IGR-IRES. We discuss potential translation initiation mechanisms for the novel ORF in the context of the IGR-IRES and 5'-adjacent stem-loop.

## Findings

**Figure 1:**
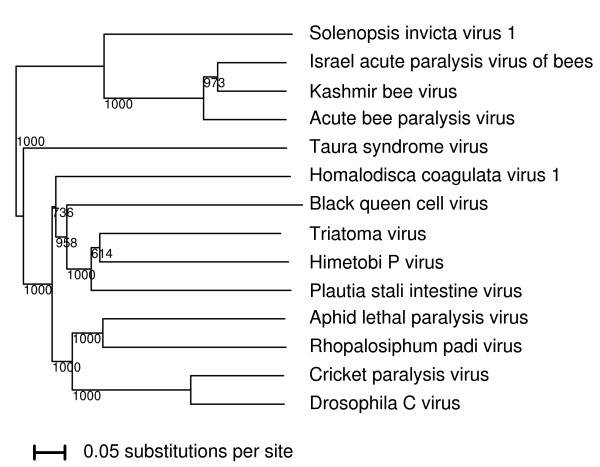
**Phylogenetic tree for representative dicistroviruses**. A simple neighbour-joining phylogenetic tree, for representative dicistroviruses based on the CDS2 (structural polyprotein) amino acid sequences. The tree was produced with CLUSTALX [18]. Columns with alignment gaps were excluded. Numbers indicate bootstrap support (out of 1000), while the scale bar represents the number of substitutions per site.

The family *Dicistroviridae *includes a number of insect- and arthropod-infecting species such as Cricket paralysis virus, Black queen cell virus, Plautia stali intestine virus and Taura syndrome virus. The species Acute bee paralysis virus (ABPV), Israel acute paralysis virus of bees (IAPV) and Kashmir bee virus (KBV) - which have been associated with Colony Collapse Disorder of honeybees - form a tight subclade within the family (Figure 1; [1-5]). The dicistroviruses have a linear positive-sense ssRNA genome containing two long ORFs. The 5' ORF (hereafter CDS1) encodes the nonstructural polyprotein while the 3' ORF (hereafter CDS2) encodes the structural polyprotein. The intergenic region (IGR) contains an internal ribosome entry site (IRES), comprising a complex and compact triple-pseudoknotted RNA structure that binds ribosomes and mediates efficient translation initation on CDS2. The IGR-IRES essentially mimics the E- and P-site tRNAs (including the P-site codon:anticodon duplex), allowing A-site initiation at a non-AUG codon, without any requirement for initiator Met-tRNA (Met-tRNA_i_) or any of the usual initiation factors (see Refs. [6-12] for recent reviews).

**Figure 2:**
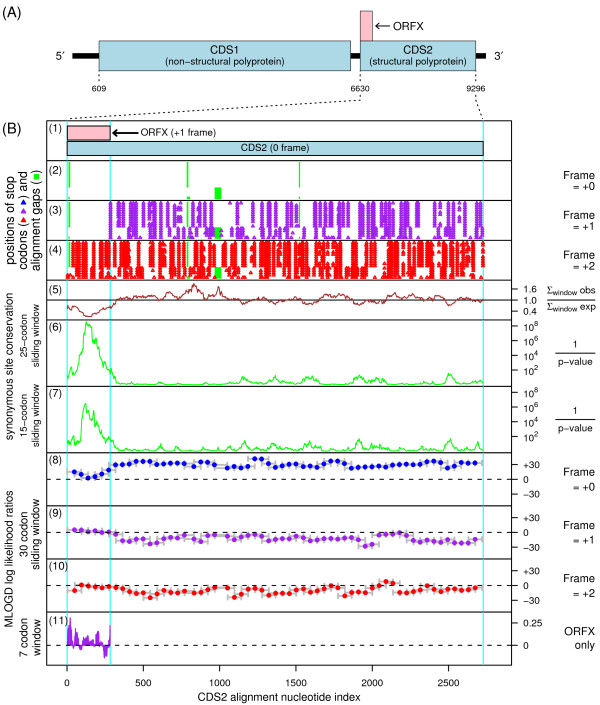
**Coding potential statistics for bee paralysis dicistrovirus CDS2 and the overlapping ORFX**. **(A) **Genome map for KBV [GenBank:NC_004807]. **(B2-B11) **Coding potential statistics based on an alignment of 16 bee paralysis virus CDS2 sequences (see Figure 3 caption for accession numbers). **(B2-B4) **Positions of stop codons in each of the three forward reading frames. Note the conserved absence of stop codons in the +1 frame within ORFX. **(B5-B7) **Conservation at synonymous sites within CDS2 (see [17]). (B6-B7) depict the probability that the degree of conservation within a given window could be obtained under a null model of neutral evolution at synonymous sites, while (B5) depicts the ratio of the observed number of substitutions within a given window to the number expected under the null model. **(B8-B10) **MLOGD sliding-window plots (see [14]). In (B8) the null model, in each window, is that the sequence is non-coding, while the alternative model is that the sequence is coding in the +0/CDS2 frame. Positive scores favour the alternative model and, as expected, there is a strong coding signature throughout CDS2 *except *where CDS2 is overlapped by ORFX. In (B9-B10) the null model is that only the CDS2 frame is coding, while the alternative model is that both the CDS2 frame and the window frame are coding. The ORFX region has consecutive positively scoring windows, albeit only just (see text; B9). **(B11) **MLOGD statistics restricted to ORFX. Here, for increased sensitivity, the null and alternative models were fitted specifically for the ORFX region.

Overlapping genes are common in RNA viruses where they serve as a mechanism to optimize the coding potential of compact genomes. However, annotation of overlapping genes can be difficult using conventional gene-finding software [13]. Recently we have been using a number of complementary approaches to systematically identify new overlapping genes in virus genomes [13-17]. When we applied these methods to the dicistroviruses, we found strong evidence for a new coding sequence - hereafter ORFX - in the bee paralysis viruses (i.e. ABPV, IAPV and KBV), overlapping the 5'-terminal region of CDS2 in the +1 reading frame (Figure 2). Here we describe the bioinformatic analyses.

Dicistrovirus sequences were extracted from GenBank, the polyprotein coding sequences were extracted, translated, aligned with CLUSTALW [18], back-translated to nucleotide sequence alignments, and clustered into separate alignments for each GenBank dicistrovirus RefSeq (using 65% nucleotide identity to the RefSeq as a cut-off threshold). Beginning with pairwise sequence comparisons, conservation at synonymous sites (only) was evaluated by comparing the observed number of base substitutions with the number expected under a neutral evolution model. The procedure takes into account whether synonymous site codons are 1-, 2-, 3-, 4- or 6-fold degenerate and the differing probabilities of transitions and transversions (see [17] for details). Statistics were then summed over a phylogenetic tree as described in [14], and averaged over a sliding window.

**Figure 3:**
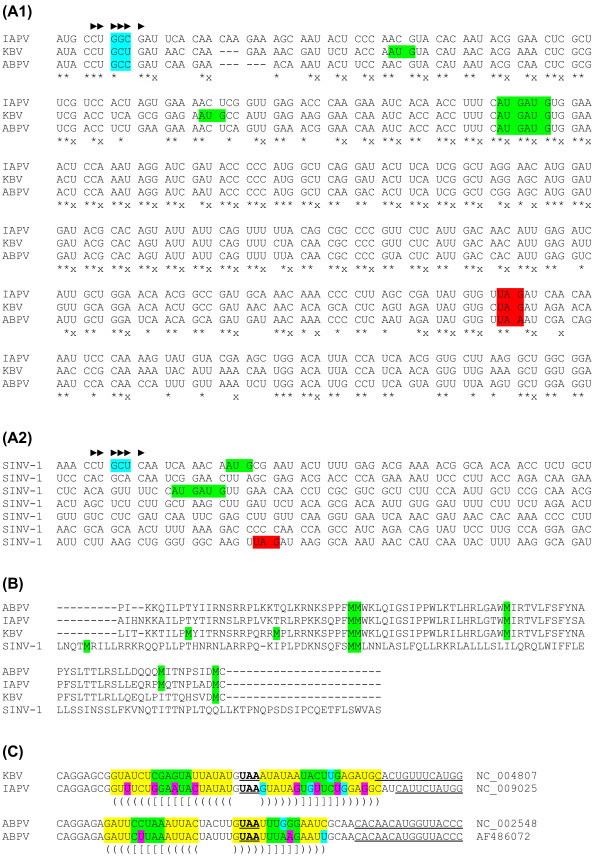
**Nucleotide and amino acid sequence alignments and predicted RNA structures**. **(A1) **Nucleotide alignment of ORFX and flanking regions for the sequences [GenBank:NC_009025] (IAPV), [GenBank:NC_004807] KBV, and [GenBank:NC_002548] (ABPV). Spaces separate +0/CDS2-frame codons. Colour coding is as follows: light blue - CDS2 IGR-IRES-mediated initiation site; red - ORFX termination codon; green - potential +1/ORFX-frame AUG initiation codons *if *ORFX is AUG-initiated (there are no intervening +0 or +2 frame AUG codons). Black arrows indicate the approximate expected initiation site if ORFX is IGR-IRES initiated (see text). Symbols '*' and 'x' represent completely conserved columns (based on a larger alignment comprising GenBank accession numbers NC_009025, EU436455, EU436456, EU436423, NC_004807, AY053375, AY053374, AY053372, AF486072, AY053367, AY053370, AY053366, AY053368, AF486073, AY053371 and NC_002548). **(A2) **The corresponding region in [GenBank:NC_006559] (SINV-1). **(B) **Amino acid alignment of the translated ORFX assuming initiation at the normal IGR-IRES initiation site but in the +1 reading frame. Methionine residues are highlighted in green. **(C) **Representative sequences showing a potential RNA hairpin structure directly upstream of the predicted IGR-IRES in the bee paralysis dicistroviruses. The CDS1 termination codons are underlined and in bold. The 5' end of the IGR-IRESs (as summarized in Ref. [11]) are underlined. Predicted base pairings are indicated by paired parentheses and coloured background shading. Substitutions that maintain the predicted base pairings are highlighted in blue (for single substitutions involving G:U pairings) or pink (for compensatory paired substitutions).

When this procedure was applied to the bee paralysis viruses (see Figure 3 caption for GenBank accession numbers), a striking and extended peak in synonymous site conservation (*p *~ 10^-14 ^for the total conservation within ORFX) was apparent at the 5' end of CDS2 (Figure 2B, panels 5-7). Such conservation peaks are indicative of overlapping functional elements, though such elements may be either coding or non-coding. However, in this case, coinciding with the conserved region there was an unusually extended and conserved absence of stop codons in the +1 reading frame (Figure 2B; panel 3), thus suggesting an overlapping coding sequence in the +1 frame as a possible explanation for the enhanced conservation. Inspection of an additional 74 sequences with only partial coverage of CDS2, but nearly complete coverage of the ORFX region, again revealed the complete absence of +1 frame stop codons in this region. If this region does not harbour an overlapping coding sequence, then the unusually high synonymous site conservation in this region almost certainly reflects some other functional element - perhaps playing some role in normal IGR-IRES initiation in the bee paralysis viruses. One possibility is simple selection against certain nucleotides in order to avoid formation of alternative RNA structures that disrupt the IGR-IRES [19]. However, the extent and degree of conservation appears unusually high (e.g. as compared with other dicistroviruses) if this is indeed the only explanation.

Next, the bee paralysis virus CDS2 alignment was analysed with MLOGD - a gene-finding program which was designed specifically for identifying overlapping coding sequences, and which includes explicit models for sequence evolution in multiply-coding regions [13,14] (Figure 2B, panels 8-11). Due to the overall high conservation, the absolute MLOGD scores tend to be low within the ORFX region (since there are fewer substitutions with which to discrimate the null or non-coding model from the alternative or coding model). Nonetheless, MLOGD predicts that ORFX is indeed a coding sequence, with consecutive positively-scoring windows in the ORFX region (Figure 2B, panels 9 and 11).

Given the location of ORFX and the unusual translation mechanism of CDS2, the translation of ORFX - if it is indeed expressed - is clearly of interest and may provide new insights into the mechanics of IGR-IRES mediated initiation. Possible ORFX translation mechanisms include (i) a portion of ribosomes initiate at more-or-less the normal IGR-IRES mediated non-Met-tRNA_i _initiation site but in the +1 frame; (ii) a portion of ribosomes, or rather 40S ribosome subunits, binding to the IGR-IRES somehow start scanning, and normal AUG-initiation takes place at a conserved tandem pair of +1 frame AUG codons ~35 codons downstream (Figure 3A) or, in some sequences, at AUG codons further 5'; or (iii) normal IGR-IRES mediated CDS2 initiation occurs but is followed by a programmed +1 frameshift into ORFX.

The synonymous site conservation plot peaks around the tandem +1 frame AUG codons (Figure 2B, panel 7; Figure 3A), falling off rapidly upstream and more slowly downstream. However, it is unclear whether or not this favours scanning and AUG initiation. There is still significant synonymous site conservation upstream of the AUG codons (Figure 3A). The peak in synonymous site conservation may just represent the region of the putative protein that is subject to the strongest amino acid constraints. The MLOGD statistics, on the other hand, indicate that the positive coding signature in the +1 frame extends right up to the 5' end of CDS2 (Figure 2B, panel 11), thus favouring the model in which a portion of ribosomes initiate at or near the usual IGR-IRES initiation site but in the +1 reading frame.

If ORFX initiation occurs at the normal IGR-IRES initiation site but in the +1 frame then translation of ORFX would result in an 11.2 kDa, 93 amino acid product in KBV, and 92 and 94 amino acid products in ABPV and IAPV respectively. If, however, initiation takes place at the downstream tandem AUG codons, then translation of ORFX would result in a 7.1 kDa, 60 amino acid product in all three species. Within the longer (i.e. 92-94 amino acid) potential ORFX product, there are 61 residues that are completely conserved between the KBV, ABPV and IAPV GenBank RefSeqs. In the region of the structural polyprotein that is encoded by the portion of the CDS2 sequence that ORFX overlaps, there are 66 completely conserved residues. Thus the putative ORFX product is apparently subject to slightly weaker functional constraints than the 'corresponding' portion of the structural polyprotein.

The IGR-IRESes of the bee paralysis viruses differ from the IGR-IRESes of most other sequenced dicistroviruses in one notable aspect - namely they have an extra hairpin structure within domain 3 (see Refs. [11,20] for details). We investigated the possibility that the presence of the extra hairpin structure might be correlated with the presence of ORFX. Two other sequenced dicistroviruses have the extra hairpin structure - (i) the ant-infecting Solenopsis invicta virus 1 or SINV-1 ([GenBank:NC_006559]; [21,22]), and (ii) the shrimp-infecting Taura syndrome virus or TSV ([GenBank:NC_003005]; [23]).

SINV-1 clusters with the bee paralysis viruses in the phylogenetic tree (Figure 1), and an analysis of its sequence shows that it does indeed contain a potential ORFX. In fact ORFX in SINV-1 is substantially longer than in the bee paralysis viruses - 125 codons if initiated in the +1 frame at the IGR-IRES normal initiation site; 83 codons if initiated at the tandem AUG codons (which are present in SINV-1 and align with the tandem AUG codons in the bee paralysis viruses); or 121 codons if initiated at an unstream intervening AUG codon (Figure 3A). (An additional SINV-1 sequence - [GenBank:FJ229495] - with partial coverage of the ORFX region contained an ORFX-frame premature termination codon [PTC] that truncates ORFX by 33 codons. However, apart from the potential for sequencing errors, PTCs in a small number of isolates are not unusual for short overlapping genes, which tend to have non-essential 'secondary' functions, and we do not believe that this ORFX-defective partial sequence necessarily precludes the presence of a functional ORFX in SINV-1.)

On the other hand, ORFX was not present in TSV. The first +1 frame AUG codon 3' of the IGR-IRES initiation site is preceded by a CDS2-frame AUG codon, and is closely followed by a +1 frame stop codon, while non-AUG +1 frame initiation at the usual IGR-IRES initiation site would only give a 16 amino acid product. Thus the presence of ORFX does not seem to correlate with the presence of the extra hairpin structure within domain 3 of the IGR-IRES.

However, we did identify a novel (so far as we are aware) potential RNA hairpin structure immediately 5'-adjacent to, but not overlapping, the IGR-IRES in the bee paralysis viruses (Figure 3C). In the KBV and IAPV RefSeqs, the hairpin comprises 18 consecutive base pairs (with a 4 nt terminal loop containing the CDS1 termination codon) and is supported by many compensatory substitutions (i.e. paired substitutions that maintain the base pairings) between KBV and IAPV. Inspection of 77 additional sequences with coverage of this region revealed six (mostly identical) sequences with single mismatches in the stem, one sequence with two mismatches, and one sequence with a 4-nt deletion at the apical end of the stem. Nonetheless, the majority of sequences retained a perfect 18 bp hairpin, and a total of 14 different substitutions that preserved the base pairings were observed. A similar, though shorter (14 bp), hairpin stucture was identified in ABPV (Figure 3C). Again, inspection of ten additional sequences revealed five different substitutions in the stem, all of which preserved the predicted base pairings. Whether the hairpin is in any way relevant to translation of the putative ORFX remains to be seen. However, preliminary experimental results indicate that presence of the predicted hairpin does have a strong effect on IGR-IRES activity (unpublished data, QS Wang and E Jan).

Recent results suggest that under certain circumstances (namely the presence of an initiator tRNA species that recognizes the P-site codon) the IGR-IRES can, at some level, mediate initiation at the P-site (presumably in competition with A-site initiation) [24]. The codon:anticodon duplex mimicking part of the IGR-IRES (a.k.a. PKI) has been shown to be dynamic and flexible [25-27], and Ref. [24] suggest that P-site initation takes place only upon dissociation of the duplex. However, this duplex is critical for selection of the CDS2 reading frame [25] so, upon dissociation of the duplex, there may be flexibility in the selection of reading frame, thus perhaps allowing +1 frame P-site initiation. In fact, all available bee paralysis virus sequences have a CUG codon at this location, which is known to be recognizable by native Met-tRNA_i _[28].

Other dicistroviruses lack a long overlapping ORF at this genomic location and lack the corresponding extended region of synonymous site conservation (data not shown). At least some other dicistroviruses do exhibit some degree of heightened synonymous site conservation at the very 5' end of CDS2, but the 3' extent of these regions appears to be much more limited than in the bee paralysis viruses (perhaps it simply reflects selection against certain nucleotides in order to avoid forming alternative RNA secondary structures that may disrupt IGR-IRES activity [19]). In fact the sequence data is rather limited for most dicistroviruses in the sense that it is difficult to make alignments with sufficiently large phylogenetically-summed diversity but sufficiently small pairwise divergences for the above analyses to produce useful statistics. Thus, there may be features in the other dicistroviruses that will remain hidden until more sequence data becomes available.

Overlapping genes are difficult to identify and are often overlooked. However, it is important to be aware of such genes as early as possible in order to avoid confusion (otherwise functions of the overlapping gene may be wrongly ascribed to the gene they overlap), and also so that the functions of the overlapping gene may be investigated in their own right. Although overlapping the structural polyprotein, there is no reason to suspect that ORFX encodes a structural protein - indeed the limited phylogenetic distribution of ORFX suggests that it does not. We are currently investigating the translation mechanism for the putative ORFX and how it relates to the IGR-IRES and the potential upstream hairpin structure.

Note: during the preparation of this manuscript, the positive coding potential of ORFX was also predicted by Ref. [29] (who name the ORF 'pog'), albeit using different bioinformatic approaches.

## Competing interests

The authors declare that they have no competing interests.

## Authors' contributions

AEF carried out the bioinformatic analysis and wrote the manuscript. All authors edited and approved the final manuscript.
